# Comprehensive Analysis of CD4^+^ T Cell Responses to CMV pp65 Antigen Restricted by Single HLA-DR, -DQ, and -DP Allotype Within an Individual

**DOI:** 10.3389/fimmu.2020.602014

**Published:** 2021-02-15

**Authors:** You-Seok Hyun, Hyeong-A Jo, Yong-Hun Lee, Sun-Mi Kim, In-Cheol Baek, Hyun-Jung Sohn, Hyun-Il Cho, Tai-Gyu Kim

**Affiliations:** ^1^ Department of Microbiology, College of Medicine, The Catholic University of Korea, Seoul, South Korea; ^2^ Department of Biomedicine and Health Sciences, College of Medicine, The Catholic University of Korea, Seoul, South Korea; ^3^ Catholic Hematopoietic Stem Cell Bank, College of Medicine, The Catholic University of Korea, Seoul, South Korea; ^4^ Translational and Clinical Division, ViGenCell Inc., Seoul, South Korea

**Keywords:** HLA-DR, HLA-DQ, HLA-DP, MHC class II alleles, CD4^+^ T cells, cytomegalovirus pp65 antigen, immunodominance, adaptive immunity

## Abstract

Within an individual, six different HLA class II heterodimers are expressed co-dominantly by two alleles of HLA-DR, -DQ, and -DP loci. However, it remained unclear which HLA allotypes were used in T cell responses to a given antigen. For the measurement of the CD4^+^ T cell responses restricted by a single HLA allotype, we established a panel of artificial antigen-presenting cells (aAPCs) expressing each single HLA allele of 20 HLA-DRB1, 16 HLA-DQ, and 13 HLA-DP alleles. CD4^+^ T cell responses to cytomegalovirus (CMV) pp65 restricted by single HLA class II allotype defined in 45 healthy donors. The average magnitude of CD4^+^ T cell responses by HLA-DR allotypes was higher than HLA-DQ and HLA-DP allotypes. CD4^+^ T cell responses by DRA*01:01/DRB1*04:06, DQA1*01:02/DQB1*06:02, DPA1*02:02/DPB1*05:01 were higher among the other alleles in each HLA-DR, -DQ, and -DP locus. Interestingly, the frequencies of HLA-DR alleles and the positivity of specific allotypes showed an inverse correlation. One allotype within individuals is dominantly used in CD4^+^ T cell response in 49% of donors, and two allotypes showed that in 7% of donors, and any positive response was detected in 44% of donors. Even if one individual had several dominant alleles, CD4^+^ T cell responses tended to be restricted by only one of them. Furthermore, CD8^+^ and CD4^+^ T cell responses by HLA class I and class II were correlated. Our results demonstrate that the CD4^+^ T cell preferentially use a few dominant HLA class II allotypes within individuals, similar to CD8^+^ T cell response to CMV pp65.

## Introduction

The diversity of human leucocyte antigen (HLA) molecules is generated by mutations, driven and maintained by purifying selection or balancing selection, such as negative frequency-dependent selection or heterozygote advantage (1–5). High allelic diversity in a population is likely to maximize the probability of survival for some individuals with protective HLA alleles against a pathogen (6–9). The association between HLA alleles and infectious disease was studied by comparing the allele frequencies of patients with a normal population (10–12). Defining how protective an allele is at the cellular level will be a physiological basis of the association studies.

CD8^+^ T cells and CD4^+^ T cells respectively recognize endogenous and exogenous antigens presented by major histocompatibility complex (MHC) class I and II molecules on antigen-presenting cells for the initiation and maintenance of adaptive immune response (13). In humans, classical MHC class I and II molecules are encoded by HLA-A, -B, and -C loci and by HLA-DR, -DQ, and -DP loci, respectively (14, 15). Most individuals are heterozygous at HLA loci and express co-dominantly up to twelve different HLA class I and II molecules. All the HLA molecules can present peptide antigen to CD8^+^ or CD4^+^ T cells, but it was not well defined whether each of these HLA allotypes is dominantly used for T cell responses to a particular antigen within individuals.

T cells respond primarily to a few among many epitopes or antigens from a pathogen (16–19). This antigen immunodominance focused on dominance among peptide epitopes of the antigen. For the enumeration of antigen-specific T cells restricted by HLA, T cells were stimulated by the peptides predicted or defined for HLA restriction (17, 20). However, determining which allele is restricted for the response is complicated by promiscuous alleles that bind multiple epitopes (21–24). MHC tetramer-based quantification can enumerate antigen-specific T cells restricted by a single HLA allele precisely, but few alleles are available (25, 26).

CMV infection is mostly asymptomatic to healthy individuals, conversely, and detrimental to immunocompromised individuals (27). For long-term persistence of adoptively transferred CMV-specific CD8^+^ T cells and protection from CMV reactivation, the presence of CMV-specific CD4^+^ T cells is known to be required (28–30). By using immune-dominant CMV pp65 that elicits mainly Th1 polarized response (31–33), we measured CD4^+^ T cell responses to CMV. We established a panel of artificial antigen-presenting cells (aAPCs) expressing a single HLA allele to enumerate the responses restricted by a single HLA allotype. Previously, allele dominance was defined that one or two alleles restricted CMV pp65-specific CD8^+^ T cell responses out of six HLA class I alleles within individuals (34). Herein, we further investigated HLA class II alleles’ dominance in antigen-specific CD4^+^ T cell responses within individuals.

## Materials and Methods

### Human Blood Samples

All healthy adult donor provided written informed consent prior to participation in this study. The participants were ranged from 29.56 ± 3.83 years of age and consisted of 5 females and 45 males. Peripheral blood mononuclear cells (PBMC) were purified by density gradient centrifugation using the Ficoll-Hypaque (GE Healthcare). PBMCs were then cryopreserved in liquid nitrogen suspended in FBS (Gibco) containing 10% dimethyl sulfoxide (Mylan) and 50% RPMI 1640 medium (Lonza). HLA typing was performed by Catholic Hematopoietic Stem Cell Bank (Seoul, Korea), using polymerase chain reaction-sequencing based typing and next-generation sequencing (MiSeq, Illumina) with genomic DNA isolated from red blood cells and granulocytes (Tiangen Biotech Corporation), as previously described (35). Research conducted for this study was approved by the Institutional Review Board of the Catholic University of Korea (MC20SESI0034).

### Cloning of HLA Class II Alleles

cDNAs of HLA-DRA allele, 21 HLA-DRB1 alleles, 10 HLA-DQA1 alleles, 11 HLA-DQB1 alleles, three HLA-DPA1 alleles, 11 HLA-DPB1 alleles were obtained from lymphoblastoid cell lines of donors (740902.50; Macherey Nagel, RT300M; Enzynomics). Because of the SNPs near start codon and stop codon, cDNAs were amplified using each primer with short consensus sequence at the coding region and the sequence at 5′ UTR, 3′ UTR with mixed bases without a change in reading frames, and cloned into pCDH lentivector (#CD523A-1; System Biosciences) without any tag using Gibson assembly (EZ015TL; Enzynomics). The sequences were verified frequently.

### Generation of Panels of aAPCs Stably Expressing Single HLA Class II Allotype

The 5×10^6^ 293TN producer cells (System Biosciences; RRID : CVCL_UL49) were co-transfected (Lipofectamine 2000; Invitrogen) with 1.3pmol psPAX2 (RRID : Addgene_12260), 0.72pmol pMD.2G (RRID : Addgene_12259) and 1.64pmol single HLA class II allele-encoding plasmid, and cultured with two days in 10ml complete media. The aAPCs, which do not express HLA class I, HLA-DR, -DQ, and -DP, were transduced with alpha chain and beta chain specific lentivirus. Specifically, HLA-DRA*01:01 only transduced monoclonal aAPCs were isolated by limiting dilution and used to determine the titer of single HLA-DRB1 allotype-specific lentivirus. The 635 ul ± 277 ul HLA-DRB1 allotype-specific lentivirus is used to transduce 1×10^4^ aAPC at an MOI of 20. The aAPCs expressing HLA-DRA*01:01 were transduced for each HLA-DRB1 allotype-specific lentivirus and expressed 67% ± 24% HLA-DR. The aAPCs expressing HLA-DQ and HLA-DP were established by transduction of 1ml alpha chain allele-specific lentivirus and 1ml beta chain allele-specific lentivirus to 1×10^4^ aAPCs.

After the transduction of each allele-specific lentivirus, HLA-DR (clone G46-6; RRID : AB_1727527) or HLA-DQ (clone Tü169; RRID : AB_2738963, Tü39; RRID : AB_395940), HLA-DP (clone B7/21; H1586; Leinco Technologies) positive aAPCs were isolated by flow cytometry-guided sorting (FACSAria Fusion, BD Biosciences). The aAPCs expressing HLA class II were cultured in RPMI 1640 supplemented with 2 mM L-glutamine,100 U/ml Penicillin-Streptomycin-Amphotericin B Mixture (Lonza), and 10% fetal bovine serum (Gibco). The aAPCs were cryopreserved after HLA class II expression was confirmed by using flow cytometry (FACSCanto, BD Biosciences).

### Peptide Pulsing

Thawed aAPCs (5 × 10^4^) expressing HLA were loaded with 60 nM of 15 amino acid peptides spanning the entire CMV pp65 protein with 11 amino acid overlap (JPT Peptide Technologies) for 3 h in serum-free media in a 96-well plate. The aAPCs loaded peptide pool were washed three times with serum-free media using centrifuge and microplate washer (405LSR; BioTek).

### Isolation of CD4^+^ T Cells

For the precise comparison of the frequency of antigen-specific CD4^+^ T cells, CD4^+^ T cells were positively isolated using magnetic microbeads (AutoMacs Pro separator; Miltenyi Biotec). The purity of CD4^+^ T cells was confirmed by flow cytometry (97% ± 2%).

### *Ex Vivo* IFN-γ ELISPOT Assay

The CMV pp65-specific CD4^+^ T cell responses restricted by a single HLA class II allotype were measured by IFNγ ELISPOT assay as described previously (34). Briefly, 100 ul of 5×10^4^ antigen-pulsed aAPCs and 5×10^5^ CD4^+^ T cells were incubated for 20h at 37°C. The spot forming cells were counted using an AID ELISPOT Reader System (AID Diagnostika GmbH). The magnitude of HLA-restricted antigen-specific CD4^+^ T cell response was calculated as [(response to aAPCs expressing HLA pulsed with peptide pools) – (response to aAPCs expressing HLA)] – [(response to aAPCs pulsed with peptide pools) – (response to aAPCs)].

### Statistical Analysis

Statistical analyses were performed using GraphPad Prism 7 software. The results were obtained from a single experiment on 45 donors. Statistical significance was determined by one-way ANOVA, Pearson’s correlation analysis, Welch’s *t*-test (with a two-tailed test of significance). Values of *P* < 0.05 were considered significant. GraphPad Prism 7, NumPy (36), FlowJo v10 (BD) were used for generating figures. Graphs are expressed as means ± standard deviation (SD) or standard error of the mean (SEM), and the sample sizes were presented in the figures.

## Results

### Establishment of aAPC Panels Expressing Single HLA Class II Allele

**Figure 1:**
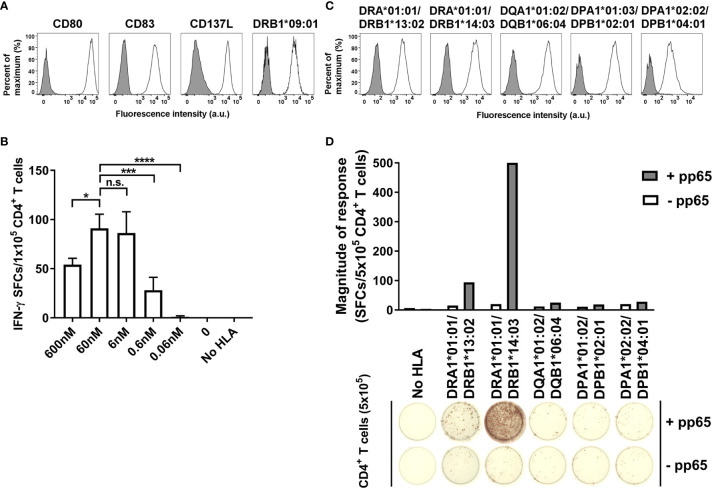
Optimization of artificial antigen-specific CD4+ T cells restricted by single HLA class II allotype. **(A)** K562 was transduced with CD80, CD83, CD137L, and DRA*01:01/DRB1*09:01 and purified by FACS to generate aAPC. **(B)** 1×10^5^ CD4^+^ T cells of HD21 were stimulated with 1×10^4^ aAPC loaded with cytomegalovirus (CMV) pp65 peptide pool at a concentration of 600 nM, 60 nM, 6 nM, 0.6 nM, or 0.06 nM, respectively. Data is presented as mean ± SDs of triplicates. n.s., not significant; *P < 0.05, ***P < 0.001, ****P < 0.0001 by one-way ANOVA. **(C)** Representative membrane expression of HLA-DR, -DQ, and -DP molecules on aAPCs expressed by HD09. **(D)** Representative IFN-γ ELISPOT assay of CD4^+^ T cells cocultured with aAPCs stably transduced with nothing (No HLA), or the HLA-DR, -DQ, and -DP alleles expressed by HD09 pulsed with nothing (-pp65) or CMV pp65 (+pp65).

To measure single HLA allotype-restricted CD4+ T cell response, K562 cell line expressing CD80, CD83, and CD137L was transduced with single HLA-DR, -DQ, or -DP allele (**Figure 1A**). The aAPCs expressing 20 HLA-DR alleles, 16 HLA-DQ alleles, or 13 HLA-DP alleles were established to cover the common alleles, which are at frequencies above 1% in Koreans. It was confirmed that these aAPCs expressed 95% or more of each allele (**Supplementary Figure S1**). The antigen-specific CD4^+^ T cells were detected high at the concentrations of 6 nM to 60 nM of pp65 peptide pool, so the concentration of 60 nM was used in subsequent studies. (**Figures 1B–D**).

**Table 1** shows the HLA class II genotypes of 45 healthy donors used in this study. Since the HLA class II is polymorphic in the alpha chains and the beta chains, an individual can express heterodimers up to four. Therefore, based on haplotype analysis, the most probable combination of alpha chains and beta chains of HLA-DQ and HLA-DP was determined and applied to this study. Since HLA-DR has very low polymorphism at the alpha chain, the most frequent allele, DRA*01:01, was used alone.

**Table 1:** Genotypes of HLA class II in 45 healthy donors.

Donor	Age	Sex	DRB1*	DRB1*	DQA1*/DQB1*	DQA1*/DQB1*	DPA1*/DPB1*	DPA1*/DPB1*
HD01	24	M	13:02	15:01	02:01/02:02	03:01/03:02	02:01/13:01	02:02/05:01
HD02	26	M	07:01	13:02	01:02/06:04	02:01/02:02	01:03/04:01	02:02/13:01
HD04	21	M	13:02	15:01	01:02/06:02	01:02/06:04	01:03/02:02	01:03/04:01
HD05	27	M	01:01	09:01	01:01/05:01	03:02/03:03	01:03/04:02	02:02/05:01
HD06	21	M	04:05	04:06	03:01/03:02	03:03/04:01	01:03/04:02	02:02/05:01
HD07	26	M	04:06	09:01	03:01/03:02	03:02/03:03	02:02/05:01	
HD08	30	M	01:01	04:05	01:01/05:01		01:03/02:01	01:03/04:02
HD09	19	M	13:02	14:03	01:02/06:04		01:03/02:01	02:02/04:01
HD10	23	M	04:07	08:02	03:01/03:02		01:03/04:02	02:02/05:01
HD11	31	M	01:01	15:01	01:01/05:01	01:02/06:02	01:03/02:02	02:02/02:02
HD12	23	M	13:02	14:01	01:02/06:04	01:04/05:02	01:03/04:01	02:02/02:02
HD14	27	M	04:06	15:01	01:02/06:02	03:01/03:02	01:03/02:01	02:02/13:01
HD16	20	M	01:01	15:01	01:01/05:01	01:02/06:02	01:03/02:01	01:03/04:02
HD17	24	M	04:06	15:01	01:02/06:02	03:01/03:02	01:03/02:01	02:02/03:01
HD18	26	M	04:06	14:05	01:01/05:03	03:01/03:02	01:03/02:01	02:02/05:01
HD19	26	M	04:05	14:05	01:04/05:03	03:03/04:01	02:02/05:01	
HD20	24	M	04:05	11:01	03:03/04:01	05:05/03:01	02:02/05:01	
HD21	32	M	07:01	09:01	02:01/02:02		01:03/05:01	02:01/13:01
HD22	27	F	07:01	09:01	02:01/02:02	03:02/03:03	01:03/02:01	02:01/13:01
HD23	29	M	13:02	14:54	01:02/06:04	01:04/05:02	01:03/02:01	01:03/04:01
HD24	31	M	04:03	14:54	01:04/05:02	03:01/03:02	01:03/04:02	02:02/02:02
HD25	26	M	04:05	12:02	03:03/04:01	06:01/03:01	02:02/05:01	
HD26	23	M	04:06	09:01	03:01/03:02	03:02/03:03	02:01/14:01	02:02/05:01
HD27	26	M	14:01	15:01	01:02/06:02	01:01/05:03	01:03/02:01	02:02/05:01
HD28	24	M	08:03		01:03/06:01		01:03/04:02	02:02/05:01
HD29	28	M	11:01	12:01	05:05/03:01		02:02/02:02	02:02/05:01
HD30	23	M	04:06	14:05	01:04/05:03	03:01/03:02	02:02/03:01	02:02/05:01
HD31	28	M	04:03	12:01	03:01/03:02	05:05/03:01	01:03/02:01	
HD33	25	M	01:01	08:03	01:01/05:01	01:03/06:01	01:03/02:01	02:02/05:01
HD34	26	M	04:04	08:03	03:01/03:02	01:03/06:01	02:02/02:02	02:01/09:01
HD35	24	M	01:01	11:01	01:01/05:01	05:05/03:01	01:03/04:02	02:02/05:01
HD37	24	M	13:02	15:01	01:02/06:02	01:02/06:09	02:02/03:01	02:02/05:01
HD38	31	M	04:05	13:02	01:02/06:04		01:03/02:01	01:03/04:01
HD39	30	M	04:05	08:03	01:03/06:01		01:03/02:01	01:03/05:01
HD40	32	M	01:01	15:01	01:01/05:01	01:02/06:02	01:03/02:02	02:02/02:02
HD41	32	M	04:04	04:05	03:01/03:02	03:03/04:01	02:01/09:01	02:02/05:01
HD42	33	M	04:10	08:03	03:03/04:02	06:01/03:01	02:01/14:01	
HD43	31	M	08:03	09:01	03:02/03:03	01:03/06:01	01:03/02:01	02:02/02:02
HD44	26	F	09:01	11:01	03:02/03:03	05:05/03:01	02:02/05:01	
HD45	25	M	07:01	12:02	02:01/02:02	06:01/03:01	02:02/05:01	
HD46	26	M	13:02	15:01	01:02/06:02	01:02/06:04	01:03/02:01	02:01/13:01
HD47	24	M	08:03	14:05	01:03/06:01	01:04/05:03	02:02/05:01	
HD48	19	F	04:01	14:54	01:04/05:02	03:03/03:01	01:03/02:01	01:03/04:02
HD49	20	F	01:01	08:03	01:01/05:01	01:03/06:01	01:03/04:02	02:02/05:01
HD50	22	M	01:01	04:01	01:01/05:01	03:03/03:01	01:03/02:01	02:02/05:01

### Population Analysis of CD4^+^ T Cell Responses According to HLA Class II Loci and Alleles

**Figure 2:**
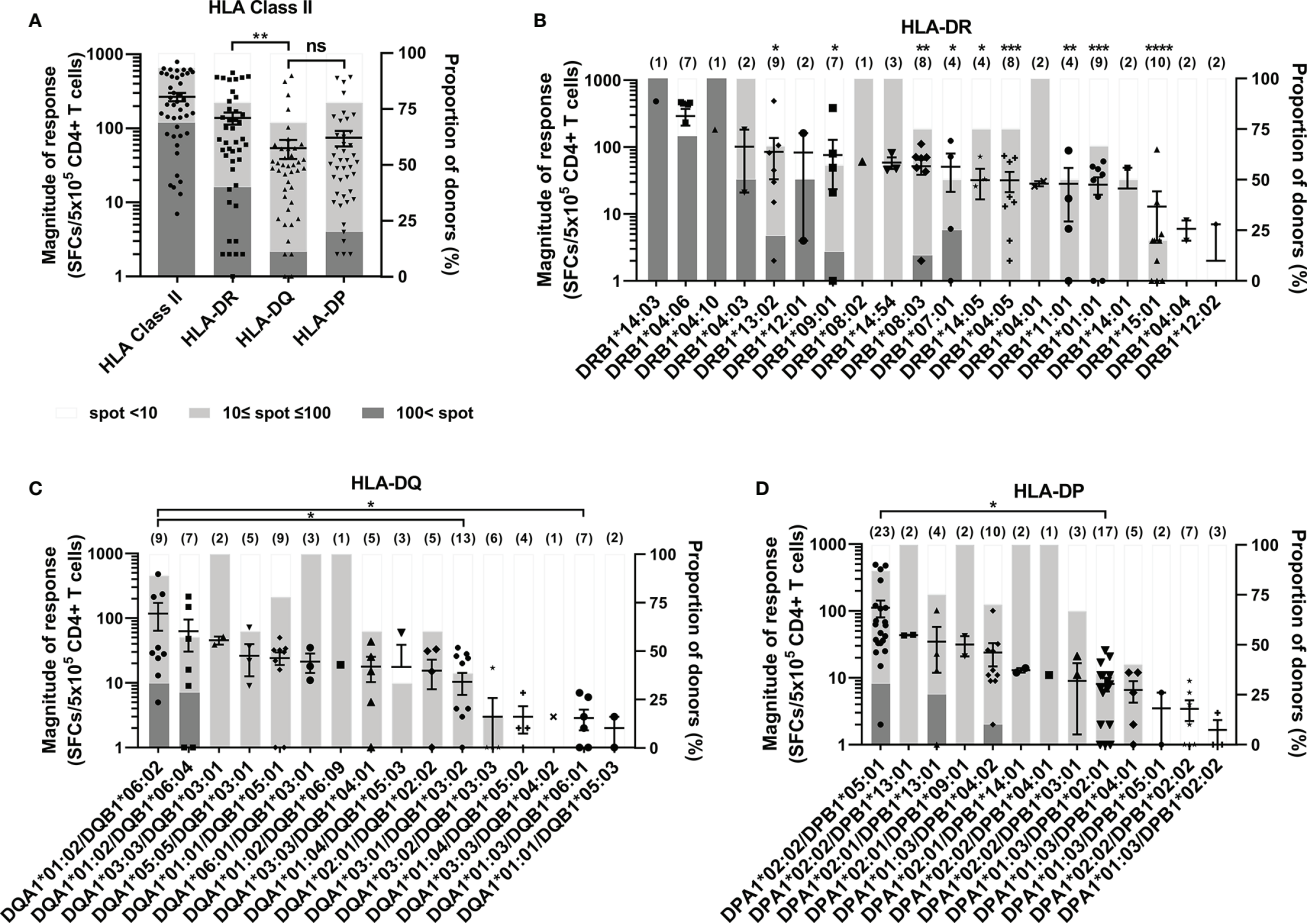
CD4^+^ T cell responses to cytomegalovirus (CMV) pp65 according to HLA class II loci and alleles. The comparison of responses by HLA-DR, -DQ, and -DP loci in 45 healthy donors **(A)**, and by HLA-DR alleles **(B)**, HLA-DQ alleles **(C)**, and HLA-DP alleles **(D)**. Each dot presents the magnitude of response by indicated allele in a donor. Error bars present mean ± SEM, and the number of donors per allele are shown in parentheses. The relative proportion of donors according to the strengths of CD4^+^ T cell responses present in stacked bar graph. Statistical analysis was performed using one-way ANOVA. ns, not significant; *P < 0.05, **P < 0.01, ***P < 0.001, ****P < 0.0001. The statistical significance with DRA*01:01/DRB1*04:06 presented above the number of donors **(B)**. DRB1 alleles are co-expressed with DRA*01:01 **(B)**. Because negative numbers cannot be shown on a logarithmic Y axis, 0 or negative values and some down vertical bars are not displayed.

**Figure 2A** shows the CD4^+^ T cell responses by each HLA class II locus, which is the sum of the responses by two alleles of each locus in an individual. The average magnitude of response restricted by each HLA class II locus was high in order HLA-DR (138 spot-forming cells (SFCs)/5×10^5^ CD4^+^ T cells) > HLA-DP (75 SFCs/5×10^5^) > HLA-DQ (54 SFCs/5×10^5^), and that by HLA-DR was significantly higher than that by HLA-DQ (**Figure 2A**, one-way ANOVA, *p* = 0.01). However, there was no significant difference between the response by HLA-DQ and the response by HLA-DP. Among positive CD4^+^ T cell responses above 10 SFCs/5×10^5^ CD4^+^ T cells, the magnitude of response above 100 SFCs/5×10^5^ CD4^+^ T cells was considered strong. The magnitude under the strong response was considered as a weak response. The proportion of donors with strong response was 40% in HLA-DR, 11% in HLA-DQ, and 20% in HLA-DP.

Among 20 HLA-DR alleles, the magnitudes of response by DRB1*04:06 were significantly higher than those of DRB1*13:02, DRB1*09:01, DRB1*08:03, DRB1*07:01, DRB1*14:05, DRB1*04:05 (**Figure 2B**). The strong response were observed in 9 HLA-DRB1, of which the proportion was high in order DRB1*14:03, DRB1*04:10, DRB1*04:06, DRB1*04:03, DRB1*12:01, DRB1*07:01, DRB1*13:02, DRB1*09:01, DRB1*08:03. The DRB1*04:04 and DRB1*12:02 did not show any positive response.

Among 16 HLA-DQ alleles, the magnitude of responses of DQA1*01:02/DQB1*06:02 were significantly higher than those of DQA1*03:01/DQB1*03:02, DQA1*01:03/DQB1*06:01 (**Figure 2C**). The DQA1*01:02/DQB1*06:02 and DQA1*01:02/DQB1*06:04 alleles only showed strong responses, of which the proportion was 33% and 29%, respectively.

Among 13 HLA-DP alleles, the magnitude of responses of DPA1*02:02/DPB1*05:01 were significantly higher than that of DPA1*01:03/DPB1*02:01 (**Figure 2D**). The DPA1*02:02/DPB1*05:01, DPA1*02:01/DPB1*13:01, DPA1*01:03/B1*04:02 alleles showed strong responses of which the proportion were 30%, 25% and 10% respectively. The average magnitude of responses by five alleles of DP^84GGPM87^, which can present endogenous antigen, was significantly lower than that by eight alleles of DP^84DEAV87^ (**Supplementary Figure S2**).

### Correlation Between HLA Class II Allele-Restricted T Cell Responses and Allele Frequency

**Figure 3:**
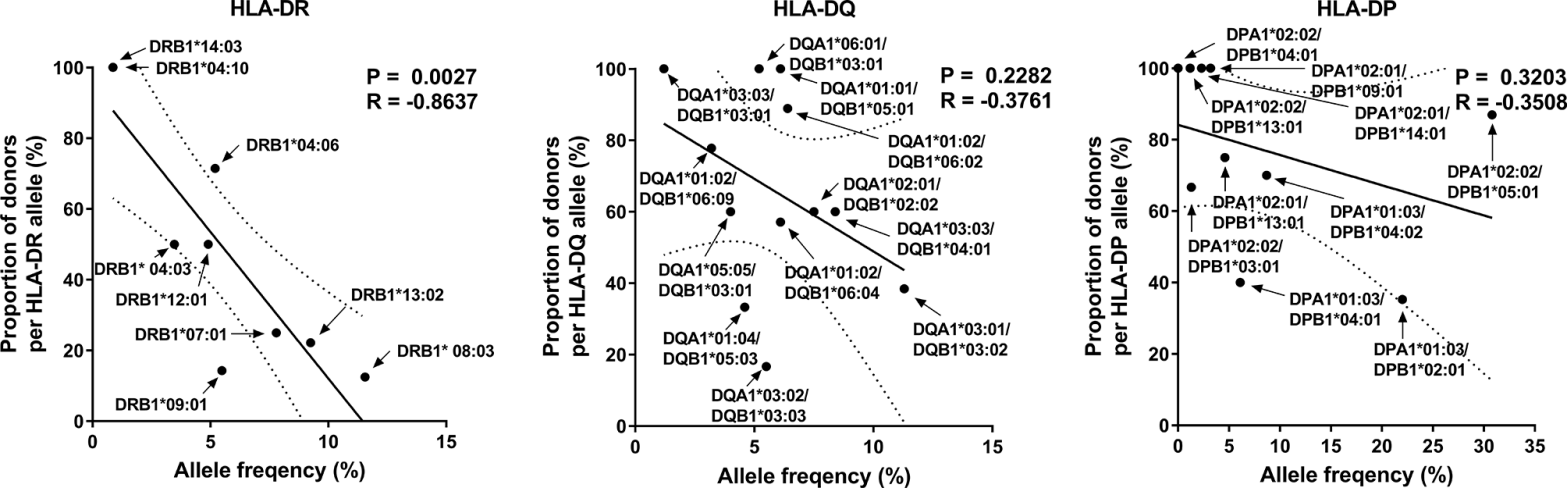
Correlation of allele frequency with the proportion of positive responses by HLA-DR, -DQ, and -DP alleles. Because the responses by HLA-DR are higher than that by HLA-DQ and HLA-DP, the proportion of donors with strong responses was compared with HLA-DR allele frequency, and that with any positive responses was compared with HLA-DQ and HLA-DP allele frequency. Statistical analysis was performed using Pearson’s correlation analysis. A line of best fit (solid lines) and the 95% confidence bands (dotted lines) were analyzed by linear regression analysis.

The diversity of MHC molecules at the population level could be driven by the magnitude of response to a given pathogen (2, 37, 38). Previously, we observed that the frequency of HLA class I alleles correlated with the proportion of responses to CMV pp65 in HLA-A and C loci (34). However, the frequency of HLA class II alleles showed a tendency of an inverse correlation with each locus’s proportion of responses. Especially, the HLA-DR locus significantly correlated inversely (**Figure 3**, Pearson’s correlation, DR *p* = 0.0027). These results suggest that the CMV infection contributes differently to HLA class I and class II diversity.

### Individual Analysis of CD4^+^ T Cell Responses According to HLA Class II Allotypes

**Figure 4:**
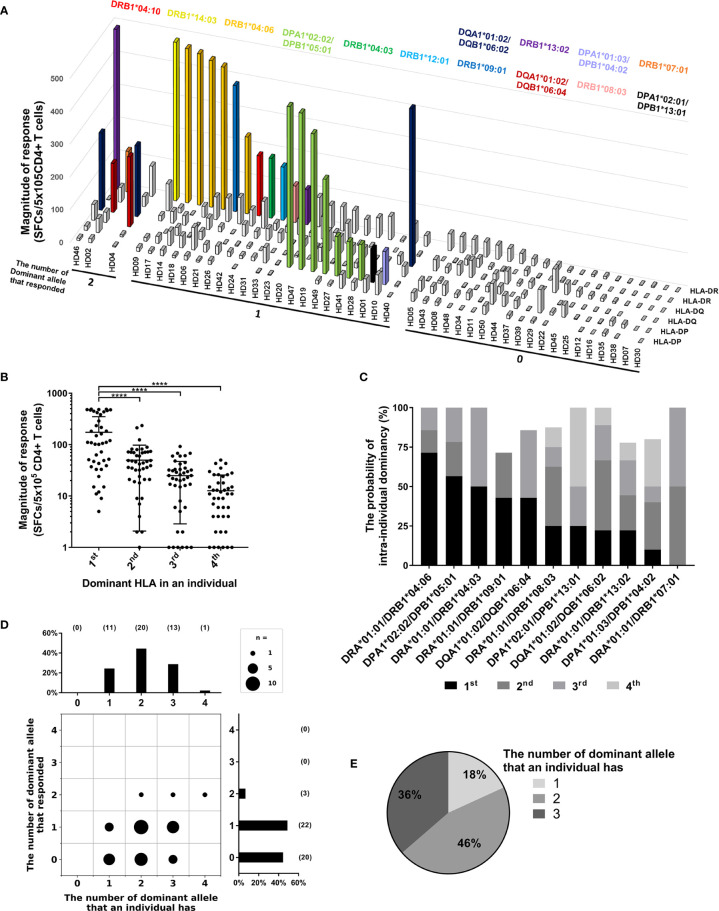
Allele dominance in CD4^+^ T cell responses within individuals. **(A)** CD4^+^ T cell responses by two alleles of HLA-DR, -DQ, and -DP loci within individuals were analyzed. Colored bars indicate the dominant alleles, which showed a strong response. **(B)** The responses within individuals were analyzed by the order of highest response by alleles of an individual. Error bars present mean ± SD. Statistical analysis was performed using one-way ANOVA. ****P < 0.0001. **(C)** The probability of intra-individual dominancy in each dominant alleles tested above three donors. **(D)** The number of dominant alleles possessed by donors (upper) and the number of strong responses by dominant alleles (right) was plotted. **(E)** In the donors who responded by one dominant allele, the distribution of the number of dominant alleles possessed by donors was analyzed.

Since up to 6 HLA class II alleles are expressed in one individual, we analyzed the number of allotypes used in CD4^+^ T cell response to CMV pp65 within individuals. In 49% of donors, only one allotype showed a strong response (**Figure 4A**). In 7% of donors, two allotypes showed a strong response. In 44% of donors, no strong response was detected. Among the donors with a strong response by one allotype, the proportion of donors with strong responses was high in the order HLA-DR, -DP, and -DQ, and the proportions were 52%, 39%, and 9%, respectively. Furthermore, across HLA class II loci, the highest response by an allotype was significantly higher than the second, third, or fourth highest response by the other allotypes within individuals (**Figure 4B**, one-way ANOVA, *p* < 0.0001). These results are the first to directly prove allele dominance in HLA class II molecules within an individual.

We speculated that the dominant alleles, which showed a strong response at least once, are not always dominant within individuals, but there is a penetrance between dominant alleles. As expected, the probabilities of the highest responses varied among the dominant alleles (**Figure 4C**). To investigate whether allele dominance was caused by the nature of the dominant allele or by competition for HLA restriction in the T cell response, the number of dominant alleles possessed by the donor and the responses restricted by dominant allotype were compared (**Figures 4D, E**). 49% of the donors showed response restricted by one dominant allotype despite possessing one to three dominant alleles (**Figures 4D, E**). 7% of the donors showed responses restricted by two dominant allotypes despite having two to four dominant alleles (**Figure 4D**). In addition, no one lacks these dominant alleles, and most of the donors have at least one dominant allele (**Figure 4D**). Collectively, there is a pattern of dominance in dominant HLA class II alleles within individuals.

### Correlation Between CD8^+^ and CD4^+^ T Cell Responses Within Individuals

**Figure 5:**
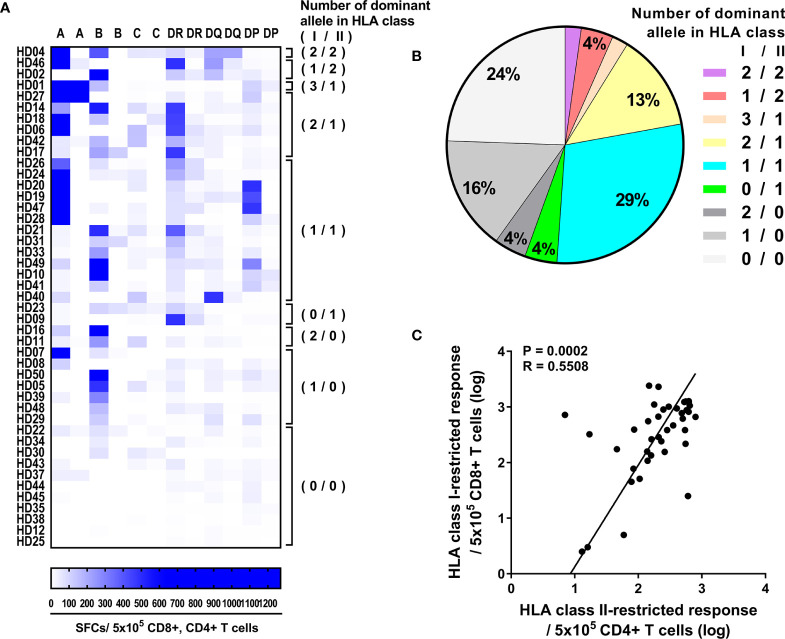
Correlation between CD8^+^ and CD4^+^ T cell responses by HLA class I and HLA class II alleles within individuals. **(A)** Allele dominance in the CD4^+^ T cell responses by HLA-DR, -DQ, and -DP and in the previously published CD8^+^ T cell responses by HLA-A, -B, and -C (34). In parentheses, the number of dominant allele in HLA class I/HLA class II that responded is presented. **(B)** The fraction of donors is plotted as the number of HLA class I/HLA class II alleles that responded strong. **(C)** Correlation between CD8^+^ T cell responses, which is the sum of the responses by HLA-A, -B, and -C alleles within individuals, and CD4^+^ T cell responses, which is the sum of the responses by HLA-DR, -DQ, and -DP alleles within individuals. The CD8^+^ and CD4^+^ T cell responses are presented as the log of the pp65 specific T cell per 5 × 10^5^. Statistical analysis was performed using Pearson’s correlation analysis. Best-fit values were analyzed by demining linear regression with 95% confidence intervals (solid line).

To extend our knowledge for HLA restriction of T cells within individuals, we compared the CD4^+^ T cell responses by HLA class II allotypes and the CD8^+^ T cell responses by HLA class I allotypes that previously defined (34). 51% of donors showed response by both HLA class I and HLA class II, and 24% of donors showed response by either HLA class I or HLA class II, and 24% of donors showed no strong response (**Figures 5A, B**). Next, the number of allotypes that showed strong responses were analyzed among the response by both HLA class I and HLA class II. One HLA class I allotype and one HLA class II allotype were dominant in 29% of donors. Two HLA class I allotype and one HLA class II allotype were dominant in 13% of donors. Among the response by either HLA class I or HLA class II, HLA class I was dominant in 20% of donors. Furthermore, CD8^+^ T cell responses by HLA class I allotypes and CD4^+^ T cell responses by HLA class II allotypes were correlated significantly within individuals (Pearson’s correlation, *p* = 0.0002) (**Figure 5C**).

## Discussion

The magnitude of CMV pp65-specific CD4^+^ T cell responses restricted by HLA-DR was higher than HLA-DQ and HLA-DP (**Figure 2**), similar to the CD4^+^ T cell response to capsid of dengue virus (17, 20). The magnitude of CMV pp65 specific CD4^+^ T cell responses and the probability of dominant responses restricted by HLA-DQ was significantly lower than HLA-DR and HLA-DP (**Figure 2A**). The low expression level of HLA-DQ explained the low magnitude of dengue virus-specific CD4^+^ T cell responses restricted by HLA-DQ (20). The combinations of HLA-DQ heterodimers competing for the antigen presentation may contribute to the low probability of dominant responses because the genes encoding α chains show high polymorphism only in HLA-DQ compared to with that of HLA-DR and HLA-DP (**Supplementary Figure S3**) (14, 39, 40). Intriguingly, the alleles of DP^84GGPM87^, which cannot bind invariant chain *via* CLIP region, present constitutively endogenous peptides (41, 42), responded significantly lower than the alleles of DP^84DEAV87^ Our data also demonstrated that alleles of DP^84GGPM87^ showed lower CMV pp65-specific CD4^+^ T cell responses than by DP^84DEAV87^ (**Supplementary Figure S2**).

Similar to HLA-DR, -DQ, -DP loci in PBMCs (20), the membrane expression of HLA-DR locus on aAPCs is significantly higher than HLA-DQ or HLA-DP locus (Mann Whitney, DR vs. DQ *p=*0.0076, DR vs. DP *p=*0.0007) (**Supplementary Figure S4A**). There was no significant difference between the membrane expression of HLA-DQ and HLA-DP (Mann Whitney, *p=*0.5551). This expression pattern was similar to the magnitude of CD4^+^ T cell responses that were also higher in HLA-DR locus than in HLA-DQ and HLA-DP locus, as shown in **Figure 2A**. We next analyzed each HLA class II allotype’s membrane expression and the average magnitude of CD4^+^ T cell responses according to the allotype (**Supplementary Figure S4B**). The membrane expression of HLA-DQ was correlated with the magnitude of CD4^+^ T cell response of HLA-DQ (Pearson’s correlation, *p =* 0.0250). The membrane expression of HLA-DR and HLA-DP were not correlated with the magnitude of CD4^+^ T cell response of HLA-DR and HLA-DP (Pearson’s correlation, DR *p=*0.5839, DP *p=*0.9674).

A possible mechanism that could explain the selection of one HLA allotype is the selection of high-affinity clones for specific epitopes. During primary CMV infection, the repertoire of T cells responding to a given viral peptide initially diverse, but at a later point in CMV infection, the repertoire of high-affinity T cells becomes more focused on a few dominant low-affinity T cell clones (43, 44). The allele dominance may be explained as the result of competition for HLA allele-restriction among T cells after infection (**Figures 4**, **5**). The two dominant allotype-restricted responses were restricted by HLA-DR and DQ, and the dominant responses restricted by HLA-DP were restricted by only one allotype within individuals (**Figure 4**). There seem to be two results by these aspects of HLA class II-restricted epitopes. *(i)* In the case of promiscuous epitopes restricted by HLA loci, T cell clones compete for a restriction between HLA alleles in an individual. *(ii)* In the case of selective epitopes restricted by specific HLA locus, T cell clones respond independently. However, since the aAPCs expressing a single HLA allotype were used, there is a limitation that the possible interaction or competition of multiple HLAs on true APC could not be observed. It is considered that cross-reactivity or competition for multiple HLA allotypes should be systematically investigated using T cell lines or TCR of dominant clones that responds to a single HLA allele (45, 46).

Among the donors who showed a strong response, the response by one of HLA class I allotypes and by one of HLA class II allotypes were dominant within individuals in most of the cases (**Figure 5**). Because endogenous antigens are presented mainly by HLA class I, and the exogenous antigens are presented mainly by HLA class II (13), so CD4^+^ T cells and CD8^+^ T cells are thought to not compete for HLA restriction with each other. In many of the following cases, the response by only one HLA class I allotype and the response by two HLA class I allotypes and one HLA class II allotype observed (**Figure 5**). Considering that the average magnitude of CD8^+^ T cell response was also higher than that of the CD4^+^ T cell response in the study of T cell responses to CMV ORFs (33), CMV-specific T cells are more selected by HLA class I than the HLA class II.

We previously reported the dominant HLA class I alleles for CMV (34). The patients with these dominant HLA class I alleles were reported to be associated with increased survival in haploidentical hematopoietic stem cell transplant (HSCT) (47). HLA-DRB1*09:01 and -DRB1*15:01 allotype showing low CD4^+^ T cell responses (**Figure 2B**) also associated with susceptibility to CMV infection after HSCT (16). A systematic investigation of allele dominance of multiple antigens may provide an immunobiological basis for the association between HLA and diseases. Measurement of allele-restricted T cell responses could be useful for predicting susceptibility against CMV infection after haploidentical HSCT and developing the bank of third-party CTL (48–50).

This study used 15 amino acid peptides spanning the entire CMV pp65 protein with 11 amino acid overlap. Naturally processed peptide fragments bound to MHC class II molecules are peptides of 13–17 amino acids (51). According to the immune epitope database (IEDB), of the 339 CMV pp65 epitopes by MHC class II in the literature, 289 CMV (85%) pp65 epitopes by MHC class II have identified 15 or less amino acid peptides (52). However, since the optimal peptide length for MHC class II affinity of approximately 18–20 amino acids has been reported in other elaborate studies (53), this point should be considered in future studies measuring CD4^+^ T cell responses. Although this study, which measures antigen-specific CD4^+^ T cell responses using ELISPOT, did not show differences due to the isolation method of CD4^+^ T cells, considering the effect of antibodies on the CD4^+^ molecule, it is reasonable to use negatively isolated CD4^+^ T cells in future studies.

In summary, our results demonstrated that CD4^+^ T cells against pp65, a major antigen of CMV, dominantly respond to one or two alleles out of six HLA class II alleles possessed by an individual, and some dominant alleles involved in this dominance. The epitope presented by the dominant alleles in the future will be identified, and alleles by which the epitope is restricted will be identified. The panel of aAPCs expressing a single HLA class I or HLA class II allotype is expected to be widely used for immunogenicity analysis of antigens associated with infectious diseases and tumors.

## Data Availability Statement

The original contributions presented in the study are included in the article/**Supplementary Material**. Further inquiries can be directed to the corresponding author.

## Ethics Statement

The studies involving human participants were reviewed and approved by the institutional review board of the Catholic University of Korea (MC20SESI0034). The participants provided their written informed consent to participate in this study.

## Author Contributions

Y-SH and T-GK conceived and designed the study. Y-SH, H-AJ, and Y-HL performed experiments. S-MK, I-CB, H-JS, and H-IC contributed reagents/materials/analysis tools. Y-SH and T-GK analyzed the data and wrote the paper. All authors contributed to the article and approved the submitted version.

## Funding

This study was supported by a grant of the Korea Health Technology R&D Project through the Korea Health Industry Development Institute (KHIDI), funded by the Ministry of Health & Welfare, Republic of Korea (HI14C3417).

## Conflict of Interest

H-JS and H-IC were employed by the company ViGenCell Inc.

The remaining authors declare that the research was conducted in the absence of any commercial or financial relationships that could be construed as a potential conflict of interest.
