# Supplementary material for: Comprehensive Analysis of CD4+ T Cell Responses to CMV pp65 Antigen Restricted by Single HLA-DR, -DQ, and -DP Allotype Within an Individual
Source: Front Immunol. 2021 Feb 15;11:602014. doi: 10.3389/fimmu.2020.602014 (PMC7917246; doi:10.3389/fimmu.2020.602014)
Supplement: Supplementary file 1 [file DataSheet_1.pdf]

Supplementary Material

Supplementary Figures

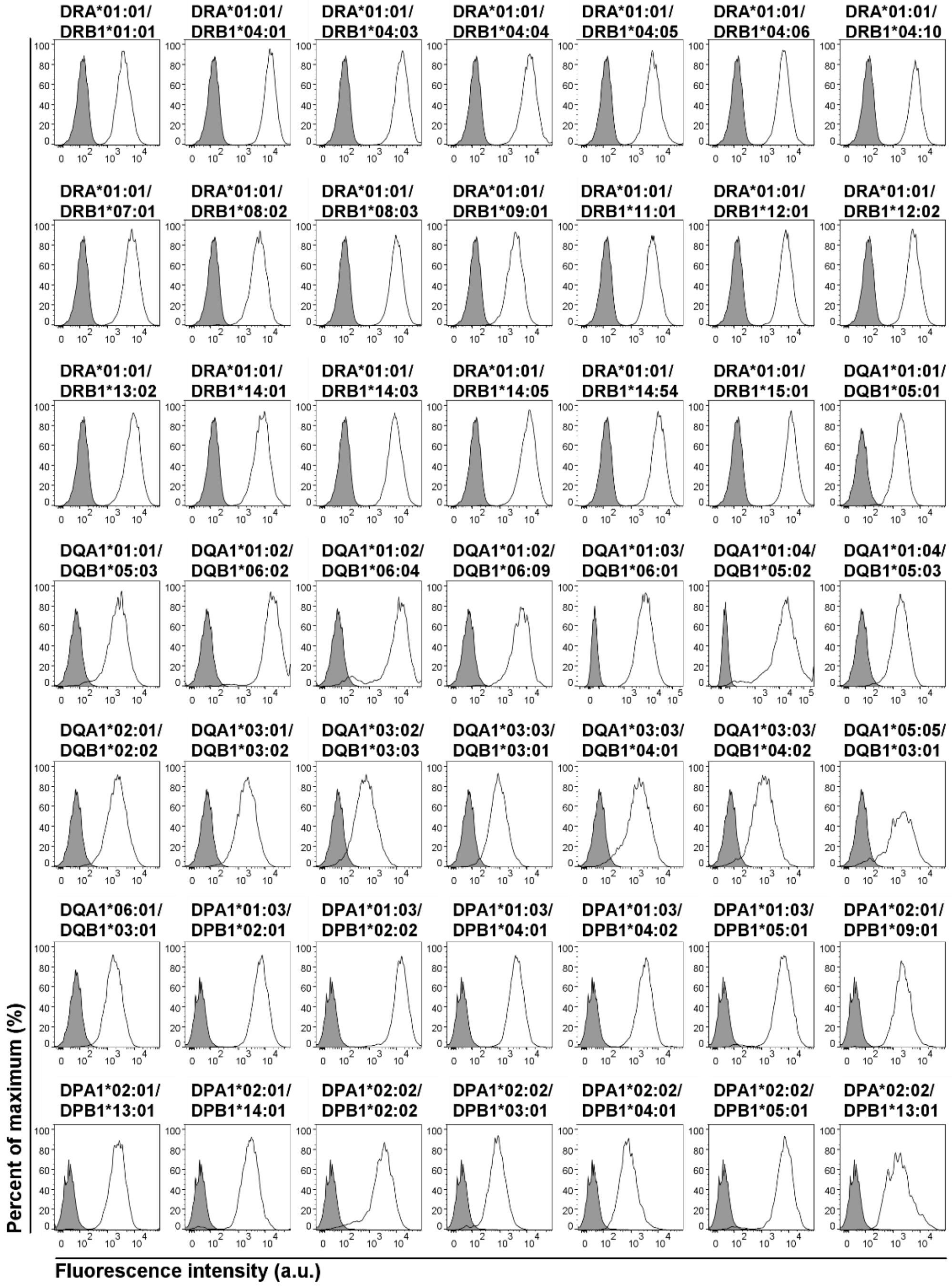

Supplementary Figure S1. Expression level of HLA-DR, -DQ, and -DP on aAPCs expressing 20 HLA-DR alleles, 16 HLA-DQ alleles, or 13 HLA-DP alleles.

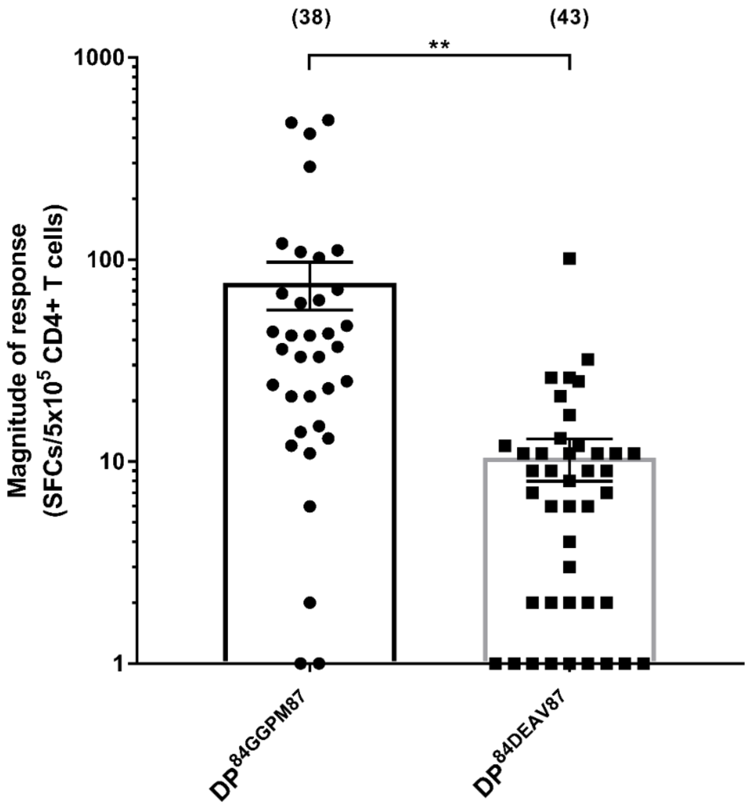

Supplementary Figure S2. The magnitude of responses restricted by DP^{84GGPM87} (DPA1*02:02/DPB1*05:01, DPA1*02:01/DPB1*13:01, DPA1*02:02/DPB1*13:01, DPA1*02:01/DPB1*09:01, DPA1*02:01/DPB1*14:01, DPA1*02:02/DPB1*03:01, DPA1*01:03/DPB1*05:01) or by DP^{84DEAV87} (DPA1*01:03/DPB1*04:02, DPA1*02:02/DPB1*04:01, DPA1*01:03/DPB1*04:01, DPA1*01:03/DPB1*02:01, DPA1*02:02/DPB1*02:02, DPA1*01:03/DPB1*02:02). Error bars present mean ± SEM, and the number of donors are shown in parentheses. Statistical analysis was performed using Welch's *t*-test. **P < 0.01.

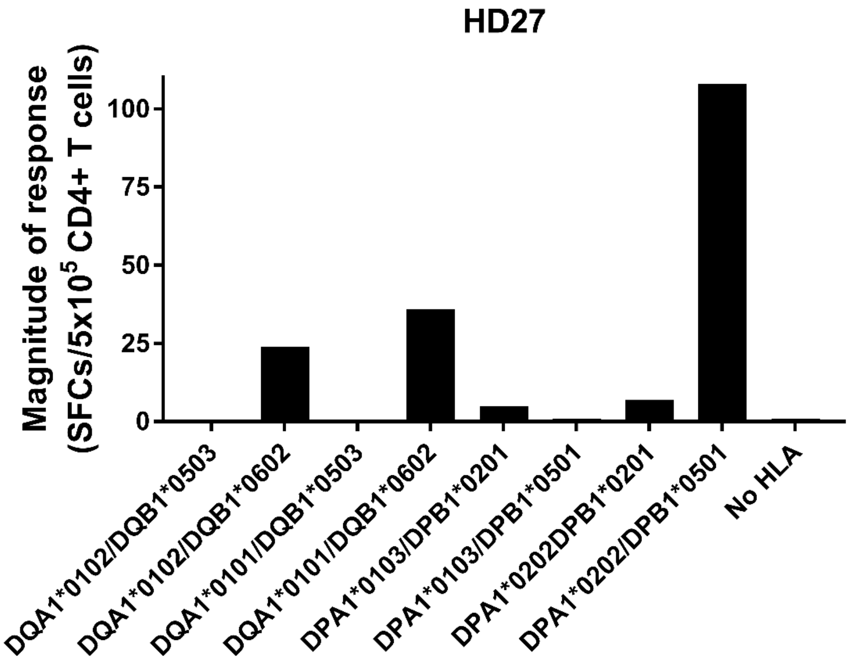

Supplementary Figure S3. The magnitude of responses restricted by four combination of HLA-DQ and -DP of HD27. The magnitude of responses was calculated as [(response to aAPCs pulsed with peptide pools) – (response to aAPCs)].

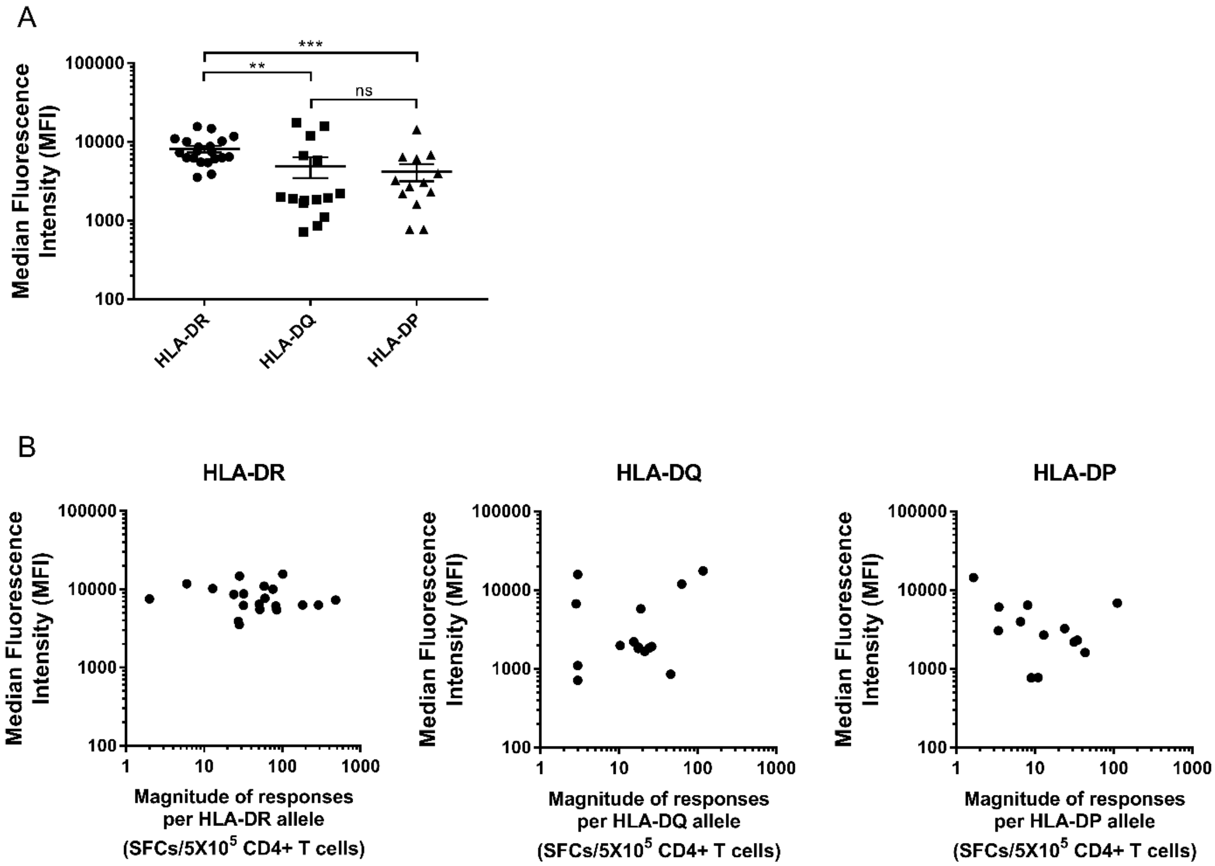

Supplementary Figure S4. The membrane expression of HLA-DR, -DQ, -DP allotypes and the average magnitude of CD4 T cell responses according to the allotypes.
